# TGF-β1 Induces an Age-Dependent Inflammation of Nerve Ganglia and Fibroplasia in the Prostate Gland Stroma of a Novel Transgenic Mouse

**DOI:** 10.1371/journal.pone.0013751

**Published:** 2010-10-29

**Authors:** David A. Barron, Douglas W. Strand, Steven J. Ressler, Truong D. Dang, Simon W. Hayward, Feng Yang, Gustavo E. Ayala, Michael Ittmann, David R. Rowley

**Affiliations:** 1 Department of Molecular and Cellular Biology, Baylor College of Medicine, Houston, Texas, United States of America; 2 Department of Urologic Surgery, Vanderbilt-Ingram Cancer Center, Vanderbilt University Medical Center, Nashville, Tennessee, United States of America; 3 Department of Pathology, Dan L. Duncan Cancer Center, Baylor College of Medicine, Houston, Texas, United States of America; New Mexico State University, United States of America

## Abstract

TGF-β1 is overexpressed in wound repair and in most proliferative disorders including benign prostatic hyperplasia and prostate cancer. The stromal microenvironment at these sites is reactive and typified by altered phenotype, matrix deposition, inflammatory responses, and alterations in nerve density and biology. TGF-β1 is known to modulate several stromal responses; however there are few transgenic models to study its integrated biology. To address the actions of TGF-β1 in prostate disorders, we targeted expression of an epitope tagged and constitutively active TGF-β1 via the enhanced probasin promoter to the murine prostate gland epithelium. Transgenic mice developed age-dependent lesions leading to severe, yet focal attenuation of epithelium, and a discontinuous basal lamina. These changes were associated with elevated fibroplasia and frequency of collagenous micronodules in collapsed acini, along with an induced inflammation in nerve ganglia and small vessels. Elevated recruitment of CD115+ myeloid cells but not mature macrophages was observed in nerve ganglia, also in an age-dependent manner. Similar phenotypic changes were observed using a human prostate epithelium tissue recombination xenograft model, where epithelial cells engineered to overexpress TGF-β1 induced fibrosis and altered matrix deposition concurrent with inflammation in the stromal compartment. Together, these data suggest that elevated TGF-β1 expression induces a fibroplasia stromal response associated with breach of epithelial wall structure and inflammatory involvement of nerve ganglia and vessels. The novel findings of ganglia and vessel inflammation associated with formation of collagenous micronodules in collapsed acini is important as each of these are observed in human prostate carcinoma and may play a role in disease progression.

## Introduction

A reactive stroma microenvironment is observed in wound repair, fibrosis, and in most proliferative diseases, including prostate cancer and benign prostatic hyperplasia. The activation of stromal cell proliferation and biology in wound repair facilitates granulation tissue formation and tissue remodeling through ECM deposition and growth factor production [1], [2]. This generalized reactive stroma response is adaptive and functions to preserve tissue integrity and homeostasis. Our earlier studies have shown that reactive stroma initiates at foci of early premalignant prostatic intraepithelial neoplasia (PIN) in human prostate gland and co-evolves with the development of carcinoma and expression of transforming growth factor beta 1 (TGF-β1) in PIN epithelium [3], [4]. Similarly, reactive stroma initiates at sites of benign prostatic hyperplasia and is associated focally with overexpression of IL-8 [5]. *In vivo* modeling studies have shown that reactive stroma promotes angiogenesis and prostate cancer progression as well as proliferative responses in normal human prostate epithelial cells, although little is understood about the specific mechanisms of reactive stroma formation [6], [7], [8]. Several growth factors have been identified as regulators of the reactive stroma microenvironment. Among these, TGF-β1 has emerged as being instrumental in both its direct and indirect effects on several pathways that mediate rapid host immune cell modulation and matrix remodeling.

TGF-β1 is a key factor released by platelets at sites of wound repair and modulates angiogenesis and inflammatory responses. The expression of TGF-β1 is elevated in most carcinomas and many proliferative diseases including benign prostatic hyperplasia, prostate cancer and prostatitis [9], [10], [11], [12]. Moreover, each of these disorders is associated with inflammation. TGF-β1 can affect several pathways known to mediate rapid host immune cell modulation, stromal biology and matrix remodeling through cytostatic, chemotactic, and fibrotic induction of different cell populations unique to stroma. However, few studies have addressed these biological responses *in vivo* using expression of active TGF-β1 in transgenic mice. Targeted overexpression in the liver in a murine transgenic model led to the development of severe cirrhosis and glomerulonephritis [13], [14]. Furthermore, targeted expression in the pancreas induced pancreatitis, whereas expression in the salivary gland resulted in glandular fibrosis and acinar atrophy [15], [16]. Overexpression of TGF-β1 in a murine MMTV model of mammary cancer demonstrated marked suppression of tumor formation [17]. However, our studies with human xenograft models have shown that TGF-β1 promotes reactive stroma formation and prostate cancer progression [18], [19]. In addition, our previous *in vitro* studies have demonstrated that TGF-β1 modulates differentiation of normal prostate stromal cells into myofibroblasts, a cell type that is unique to tissues undergoing fibrosis, remodeling, or reactive stroma formation in several disorders [4].

Accordingly, TGF-β1 is emerging as a potential target of therapeutics in modulating fibrosis, inflammation, and tumor progression. It is clear that the pleiotropic nature of TGF-β action is due in part to the local cell and tissue-specific milieu. Therefore, we sought to address the potential etiological role of aberrant TGF-β1 action in inducing stromal microenvironment responses in normal intact murine prostate gland tissue. We report here a transgenic mouse model constructed with epitope tagged and constitutively active TGF-β1 expressed in the prostate gland epithelium. In addition, we have evaluated a tissue recombination model generated with human prostate epithelial cells engineered to overexpress active TGF-β1 and stromal cells in xenografts. Results reported here demonstrate that expression of constitutively active TGF-β1 results in age-dependent phenotypic alterations characterized by attenuation of secretory acini walls, induction of fibroplasia, and inflammation in vessels and nerve ganglia. We also report the formation of unique fibrotic collagenous micronodules, shown previously to be associated with human prostate cancer.

## Materials and Methods

### Ethics Statement

All animals were handled in strict accordance with good animal practice as defined by the relevant national and institutional animal welfare bodies, and all animal work was approved by IACUC protocol AN-1867.

### Transgenic construction

The composite probasin promoter ARR2PB (provided by Dr. Robert J. Matusik) was used to target transgene expression specifically to murine prostate epithelium as reported previously [8], [20], [21]. ARR2PB was cloned 41 bp upstream of the HA-tagged TGF-β1(a) insert in the pEF6 vector (Courtesy of Dr. Larry Wolfraim, NCI, NIH) that also contained a 3′ BGH polyA tail [22]. The resulting 2.7 kB ARR2PB-HA-TGF-β1(a) DNA fragment was excised, gel purified, and provided to the Baylor Genetically Engineered Mouse Core for microinjection into C57BL/6 mouse egg pronuclei. The HA-TGF-β1(a) insert was also excised from pEF6 and cloned into the pBMN-IRES-eGFP retroviral vector, packaged and virus used to transduce LNCaP human prostate carcinoma cells and NHPrE1.2 human prostate epithelial cells as reported previously [19], [23]. Expression of full length HA-TGF-β1(a) protein in conditioned media was verified by Western blot to HA epitope and ELISA to TGF-β1 (data not shown).

PCR amplification of tail-derived genomic DNA was used to identify transgenic mice. Primers designed to amplify a 1.2 kb region of transgene encompassing a region upstream of the start codon to the internal HA tag were as follows: 5′ CAGTGTGGTGGAATTGCCCTTATCTGGTACC and 5′CAGAGATGCTAGTCTGGCACGTCGTATGGGTAGCT. Amplification of MbCx7 served as an internal positive control using the following primers: 5′ GATGTGCTCCAGGCTAAAGTT and 5′ AGAAACGGAATGTTGTGGAGT. PCR reactions used Platinum *Taq* DNA polymerase (Invitrogen) and 2 mM MgCl_2_ for 32 cycles at a 55°C annealing temperature for 1 min. Five founder mice were transgenic and bred for examination of F1 generation. Screening of F1 mice revealed that four founders exhibited germline transmission.

### Animal husbandry

All housing and manipulations of mice followed an approved IACUC protocol (AN-1867). Male and female mice from the F1 generation were bred with wild-type mates and resulting F2 generation males were screened by PCR of genomic tail DNA. Wildtype and transgenic littermates from each line were examined for RNA production by RT-PCR. Primers designed to amplify a region encompassing the internal HA tag were as follows: 5′ATACCAACAGCTACCCATACGACG and 5′ CACTTCCAGCCCAGGTCC. GAPDH primers designed to anneal to endogenous genetic material were as follows: 5′ CCTACCCCCAATGTGTCCG and 5′ CCTTCTTGATGTCATCATACTTGGC. Reverse transcription was performed at 50°C for 30 min followed by PCR amplification for 28 cycles at a 60°C annealing temperature for 1 min.

### Cell lines

LNCaP human prostate carcinoma cells were acquired from ATCC (American Type Culture Collection, Manassas, VA) and maintained in RPMI 1640 media (Invitrogen, Carlsbad, CA) supplemented with 10% fetal bovine serum (FBS) (Hyclone, Logan, UT), 100 units/ml penicillin, and 100 μg/ml streptomycin (Sigma, St Louis, MO). The Phoenix A packaging cell line was purchased from ATCC and maintained in DMEM with high glucose (Invitrogen) supplemented with 10% heat inactivated FBS (Hyclone), 2 mM glutamine (Invitrogen), 100 units/ml penicillin, and 100 μg/ml streptomycin (Sigma). The C57BL/6 mouse prostate stromal cell line was generated as reported previously [19].

To engineer cell lines, primers were designed to amplify cDNA from the pEF6-HA-pTGFβ1(a) vector for cloning into the multicloning site of the pBMN-Ires-eGFP retroviral vector (courtesy of Dr. Gary Nolan). Sequences were verified to confirm construct fidelity and pBMN-HA-TGF-β1(a)-IRES-eGFP was then transfected into Phoenix A cells with a Calcium Phosphate Transfection kit (Invitrogen) for retroviral engineering of the NHPrE1.2 human prostate epithelial [24] and LNCaP cell lines. Virus was collected, filtered through 0.45 μm and applied to infect cells as described previously [19]. Infected cells were subsequently FACS sorted by GFP expression.

### Kidney capsule xenografts

Tissue recombination was performed as described previously [24]. Briefly, urogenital mesenchyme was dissected from 18dpc rat pups and recombined with 6×10^5^ NHPrE1.2 cells (engineered for TGF-β1 expression or vector control) in 50 μl collagen. Collagen plugs were then grafted under the kidney capsule of SCID mice for 8 or 12 weeks and removed for formaldehyde fixation and paraffin embedding. Sections were cut at 5-micron thickness for immunostaining.

### Immunohistochemistry

The intact urogenital tract including the bladder, seminal vesicle and prostate gland was removed en mass, fixed in 4% paraformaldehyde and processed for histology and immunohistochemistry as we have reported previously [8]. For initial orientation and evaluation of phenotype, 25 serial sections were generated and sections 8 and 16 were stained with hematoxylin and eosin (H&E). All immunostaining was performed on adjacent serial sections with the MicroProbe Staining System (Fisher Scientific), and counterstained with hematoxylin as described previously [4], [8]. To validate protein expression by transgene in murine ventral prostate epithelia via the HA epitope tag, sections from a cohort of transgenic and control mice were subjected to enzymatic antigen retrieval done by incubation in 0.1% pronase (Calbiochem) at 50°C for 3 min followed by blocking with goat serum (5%) for 30 min at RT. IHC was performed with rabbit anti-HA (1∶100, Santa Cruz, sc-805) overnight at 4°C, followed by biotin-conjugated goat anti-rabbit secondary antibody (1∶500, Molecular Probes, D-20691) for 1 hr at 37°C. For tenascin-C immunostaining, representative tissue was subjected to pronase antigen retrieval as described above, followed by a rabbit anti-tenascin-C antibody (1∶50, Chemicon, AB19013) overnight at 4°C and secondary antibody as described above for anti-HA. All other IHC was performed on representative tissues subjected to heat-mediated citrate (pH 6) antigen retrieval followed by blocking with goat serum (5%) for 30 min at RT. Primary antibodies were rabbit anti-CD115 (1∶50, Abcam, ab61137); rat anti-F4/80 (1∶100, Abcam, ab6640); rabbit anti-collagen type IV (1∶500, Abcam, ab19808); and rabbit anti-p65 (1∶200, Santa Cruz, sc-109). Each was incubated overnight at 4°C, followed by biotin-conjugated, goat-anti-rabbit secondary antibody (1∶500, Molecular Probes, D-20691) or biotin-conjugated, goat-anti-rat secondary antibody (1∶500, Molecular Probes, D-20697) for 1 hr at 37°C. Primary antibodies for IHC on tissue recombinants were rat anti-F4/80 (1∶200, Serotec, MCA497GA) and rabbit anti-p65 (1∶200, Santa Cruz, sc-109). Sections were incubated overnight at 4°C, followed by biotin-conjugated, goat-anti-rat (1∶200, Zymed, 81-9540) or swine-anti-rabbit (1∶200, Dako, E0353) secondary antibodies, respectively.

### Evaluation of tissue phenotype

Ventral prostate and surrounding regions were evaluated as the initial focus of this analysis due to the well-characterized simple epithelial acini histology and stroma of this prostate lobe. Tissues from each respective transgenic line were evaluated separately and data grouped together for comparison with wild-type littermates. Phenotypic changes in inflammatory foci, epithelial wall attenuation (thinning), and stromal fibroplasia were each evaluated as separate histopathologic parameters using a semi-quantitative protocol similar to what we have reported previously [25]. H&E sections (section number 8 and 16) of prostate tissue from all mice listed in Table 1 (n = 41 transgenic mice, n = 38 control mice) were imaged (200× and 400× fields) and graded for each parameter separately according the following pathologic scale: 0 = wild-type phenotype; 1 = minor phenotypic involvement (non-wildtype histology and minor involvement in less than 10% of the tissue or region being evaluated); 2 = moderate phenotypic involvement (moderate involvement in 10–50% of the tissue or region being evaluated); 3 = severe phenotypic involvement (major involvement in >50% of the tissue or region being evaluated). Inflammatory foci were defined as focal sites of accumulation of lymphocytes and granulocytes. Epithelial wall attenuation was defined as epithelial walls thinned to less than 50% of wild type epithelial height. Fibroplasia was defined as foci of stromal hyperplasia following standard pathological criteria and accumulation of prototypical reactive stroma. Values were averaged for wildtype and transgenic mice and divided into categories of less than 1 year and greater than 1 year of age.

**Table 1:** Timepoints of phenotype analysis.

Age Range (weeks)	Wild-Type (n)	Transgenic (n)
10–29	7	9
30–49	11	15
50–119	12	11
120–149	8	6

(n = number of transgenic and wild-type animals evaluated in each age range).

### Statistical evaluation

Mann-Whitney and non-parametric Student's *t*-tests were used to assess significant differences for each measured phenotype. Significance of the frequency of collagenous micronodules was evaluated using Fisher's exact test. For quantitative evaluation of CD115 and F4/80 staining, positive cells were counted manually in the nerve ganglia in a blinded study (reviewer blinded to the mouse genotype). Values were averaged for each mouse and Kruskal-Wallis non-parametric ANOVA and Mann-Whitney tests were used to determine significance. For all tests, data generating a *P* value less than 0.05 was considered statistically significant. All analyses were performed using the Prism statistical software package.

## Results

### Targeted HA-TGF-β1(a) Expression to Mouse Prostate Gland

**Figure 1:**
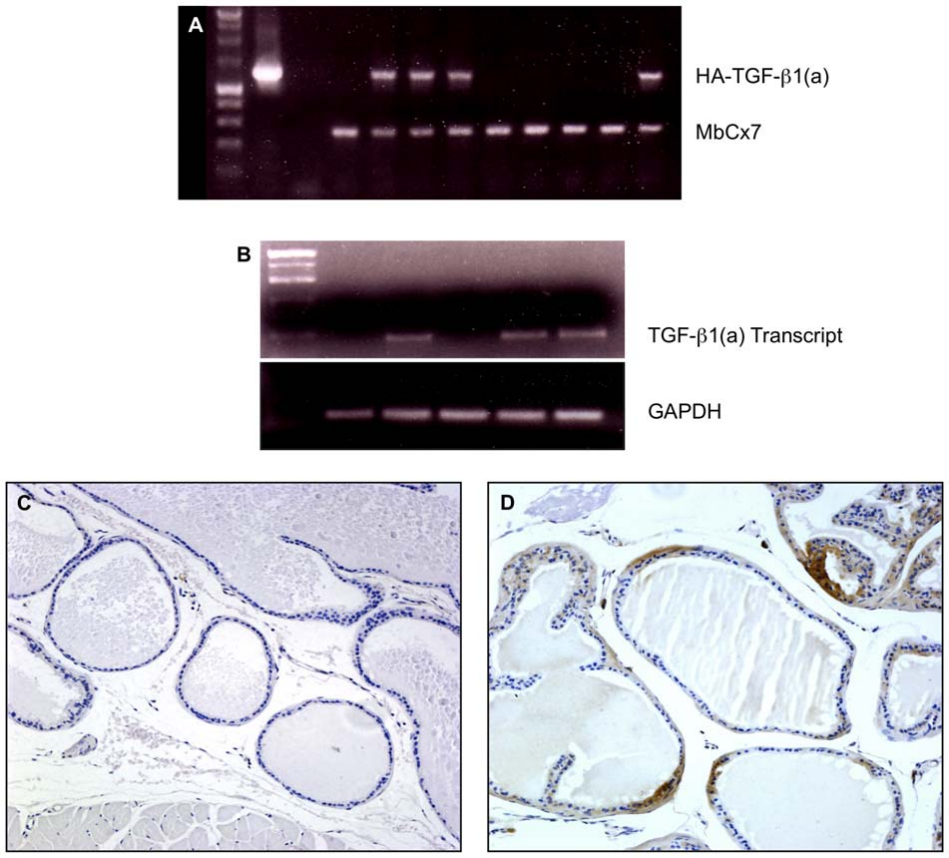
HA-TGF-β1(a) transgenic expression. ***Panel A*** **.** Representative genomic DNA PCR screen of transgenic litters demonstrating germline transmission. ***Panel B.*** RT-PCR for transgene message in the prostate gland. GAPDH amplification serves as an internal control. ***Panel D.*** IHC of transgene demonstrates focal expression in ventral, lateral, and dorsolateral regions of secretory acini. ***Panel C.*** Wildtype acini demonstrate no immunoreactivity to HA epitope. Images C and D were captured at ×200 magnification.

Expression of constitutively active TGF-β transgene was targeted to epithelial cells of the male urogenital tract in order to assess TGF-β1 actions on the stromal environment. An HA-TGF-β1(a) construct was used containing mutations in Cys223 and Cys225 to provide constitutive biological activity and an internal HA epitope tag as previously reported [22]. To verify biological activity of recombinant protein expressed from this construct, prostate epithelial cells *in vitro* were engineered to express HA-TGF-β1(a) construct. Expression and secretion of full-length protein was confirmed by Western blot (HA epitope) and ELISA for TGF-β1 protein (data not shown). Biological activity of secreted protein was confirmed via induction of phenotypic changes in C57BL/6 murine prostate stromal cells as we have reported previously [23]. To target expression in transgenic mice, the murine enhanced probasin promoter (ARR_2_PB) was used to drive HA-TGF-β1(a) expression in the prostate gland [20]. Four lines of transgenic mice were confirmed by PCR screening tail DNA for genomic integration (Figure 1A). Transgene mRNA was detected in the prostate gland of all four lines with three of the four transgenic lines exhibiting higher levels (Figure 1B). Transgenic mice exhibited no apparent alterations in development of urogenital tract or reproductive potential relative to control mice.

Age-matched male reproductive tract was examined in all four transgenic lines and control littermate mice from 12 weeks to 144 weeks of age (n = 38 control and n = 41 transgenic mice) as outlined in Table 1. HA-TGF-β1(a) protein was expressed in a focal manner in the ventral, dorsal, and lateral prostate gland as shown in Figure 1D. Age-matched wild-type mice showed no specific immunoreactivity for HA epitope tag (Figure 1C). As expected, focal expression of HA-TGF-β1(a) was particularly evident in the ventral prostate lobe in transgenic mice owing to the efficacy of ARR_2_PB induced expression in this region of murine prostate gland. All transgenic lines exhibited similar phenotypic changes in epithelium and stroma.

### Expression of HA-TGF-β1(a) induces an age-dependent attenuation in epithelial acini wall integrity

**Figure 2:**
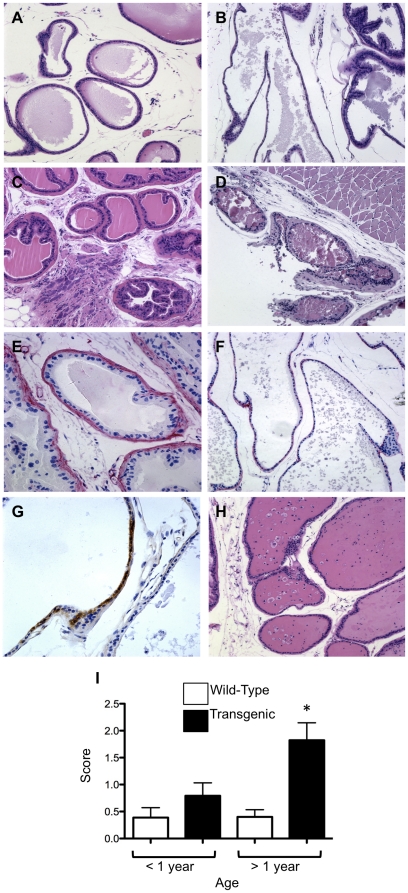
Expression of HA- TGF-β1(a) results in attenuation of prostate gland secretory epithelium. ***Panels A and C.*** Representative micrographs of wild-type ventral prostate from 30-week and 62-week old mice, respectively. Micrographs shown are representative of all ages examined. ***Panel B.*** Overexpression of TGF-β1 results in thinning and denuding of the epithelial wall in a 29-week old transgenic mouse. ***Panel D.*** Ventral prostate from 62-week old transgenic mouse demonstrates severe attenuation. ***Panel F***. IHC for collagen type IV demonstrates discontinuity of basement membrane in transgenic compared to wild-type (***Panel E***) prostate. ***Panel G.*** HA immunolocalization is observed in some areas of wall attenuation in transgenic mice. ***Panel H.*** Pyknotic appearing cells are evident in the lumen of acini in transgenic mice. ***Panel I***. Quantitation of epithelial attenuation yields significantly greater thinning in aged transgenic compared to wild-type ventral prostate gland (*P*<0.05). Images A–F and H captured at ×200 magnification. Image G captured at ×400 magnification.

Initial analyses suggested an alteration in the epithelial wall integrity in transgenic mice as compared with control mice (Figure 2). Some acini exhibited foci of attenuated epithelium, evident as diminished cell height and acinar wall thickness in mice as early as 19 weeks of age (Figure 2B). In some foci, epithelium exhibited a flattened, near squamous morphology (Figure 2D). Consistent with this, a more irregular spacing pattern and density of nuclei in the epithelial wall was initially observed at 30 weeks of age. These changes were associated with a higher frequency of pyknotic intraluminal cells in transgenic mice, suggesting cell apoptosis (Figure 2H). Of interest, the severity and frequency of this response was particularly apparent in transgenic mice over a year of age (Figures 2D and 2F). In contrast, acinar walls in control mice exhibited a prototypical cuboidal epithelium with a continuous epithelial height and regular linear spacing of nuclei at all ages examined (Figures 2A and 2C). Alteration in acini wall thickness was associated with focal discontinuity and thinning in the basal lamina as shown by IHC for basal lamina collagen IV (Figures 2E and 2F). In addition, IHC for the HA epitope tag showed that focal expression of transgene could be observed in spatial association with regions of attenuated epithelial wall acini (Figure 2G). Quantitation of changes in epithelial acini wall thickness demonstrated significant differences in transgenic mice over a year of age compared to aged wildtype controls (Figure 2I).

### Expression of HA-TGF-β1(a) in prostate epithelia induces an inflammatory response

**Figure 3:**
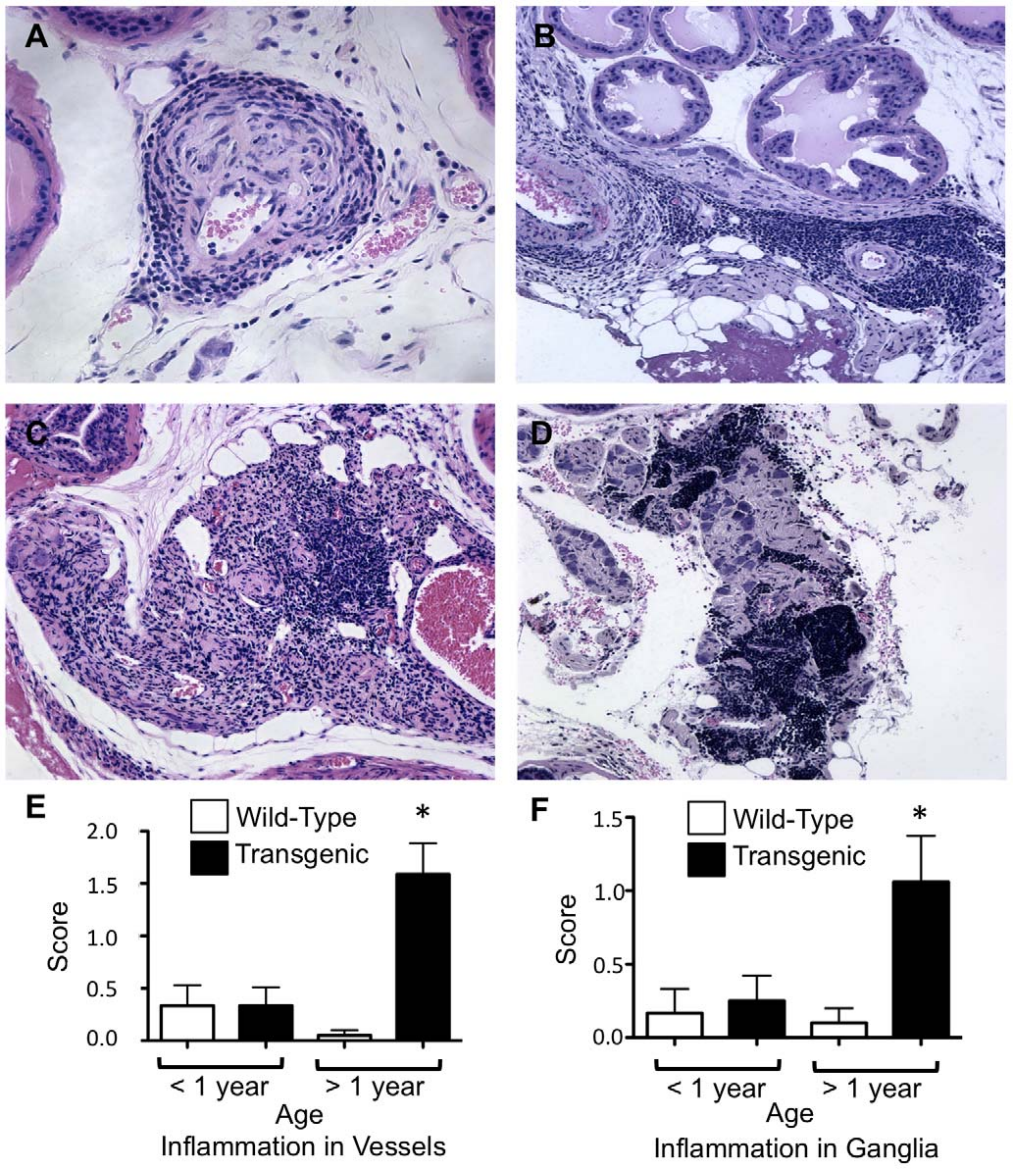
Inflammation is increased in an age-dependent manner in nerve ganglia and associated vasculature of TGF-β1 transgenic mice. ***Panel A.*** Vascular fibroplasia is evident in the wall of some vessels. ***Panel B.*** Focal regions of inflammation characterized by accumulations of immune cells is observed adjacent to some vessels in an age-dependent manner. ***Panel C and D.*** Peripheral inflammation appears within most prostatic interlobar parasympathetic ganglia and neurovascular bundles in transgenic mice. ***Panels E and F***. Quantitation yields significantly greater inflammation in aged transgenic compared to wild-type vessels and ganglia, respectively (*P*<0.05). All images captured at ×200 magnification.

Concomitant with alterations in epithelial wall integrity, transgenic mice also exhibited focal regions of inflammation in the vasculature and local parasympathetic ganglia. Vascular fibroplasia typified by a thickened tunica media in intermediate vessels was noted in some transgenic mice and was usually associated with inflammation in the vessel wall (Figure 3A). Foci of inflammation were also observed adjacent to vessels beginning at 34 weeks in transgenic mice (Figure 3B). Of interest, the most highly penetrant phenotype in transgenic mice was inflammation in the nerve ganglia associated with interlobular neurovascular bundles in transgenic mice over a year of age (Figures 3C and 3D). Nearly all ganglia in this cohort of transgenic mice were infiltrated with immune cells, whereas age-matched control mice generally exhibited little inflammation in ganglia. Similar to patterns of acini wall attenuation, independent grading showed significant increases in inflammation of the vessels and ganglia in transgenic mice over 52 weeks of age compared to either age-matched wildtype mice or to mice under one year of age (Figures 3E and 3F).

### Expression of HA-TGF-β1(a) in prostate epithelia induces focal fibroplasia and nodular stromal lesions

**Figure 4:**
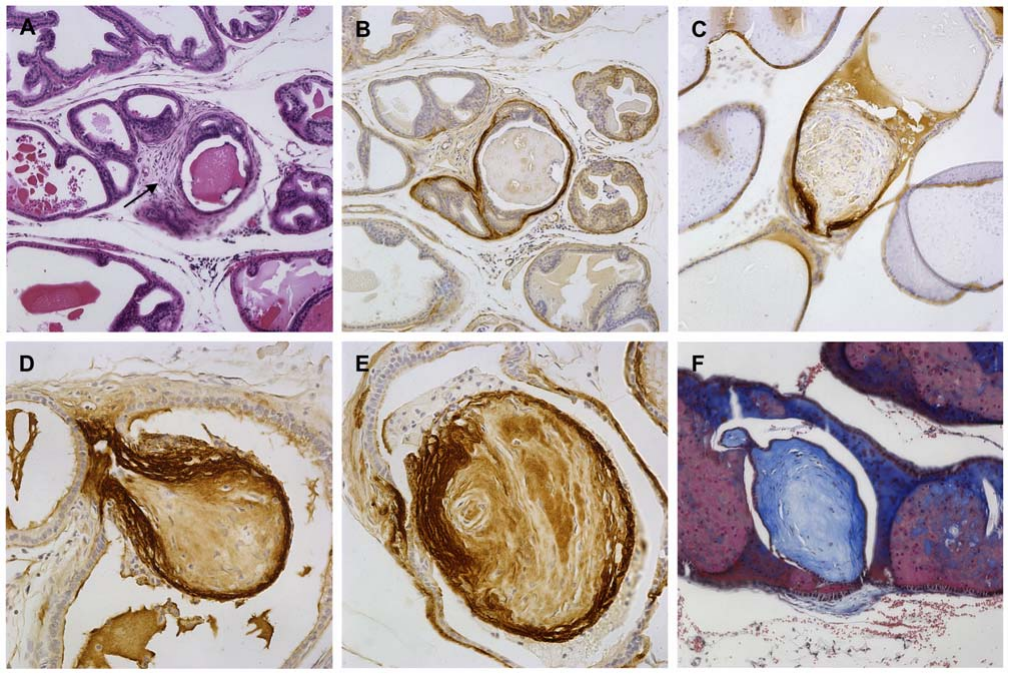
Fibroplasia in transgenic mice exhibits markers of reactive stroma. Serial sections showing stromal thickening (***Panel A***, arrow) with increased deposition of tenascin-C (***Panel B***, IHC for tenascin-C), consistent with a reactive stroma phenotype in areas surrounding some attenuated epithelial acini. ***Panel C***. Tenascin-C immunoreactivity is also deposited near the base of stromal micronodules. ***Panel D and E***. Tenascin-C deposition appears to form lamellar layers near the base and periphery of stromal micronodules. ***Panel F***. Masson's trichrome staining indicates nodules are composed of collagen (blue). Images A–D & F were captured at ×200 and image E at ×400 magnification.

**Figure 5:**
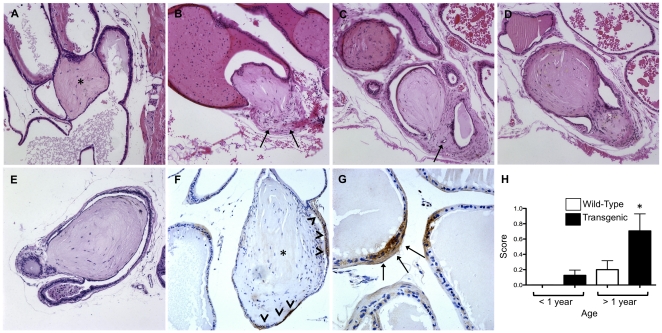
Collagenous micronodules in TGF-β1 transgenic mice. ***Panel A*** **.** Micronodules originate from the stroma immediately adjacent to epithelial acini (asterisk). ***Panel B***. Micronodules are associated with a fibroplastic reactive stroma at their base (arrows) and project into the wall of epithelial acini. ***Panel C.*** Periacinar stroma is thickened in regions adjacent to micronodules. Vessels are often observed at the base of stromal micronodules (arrow). ***Panel D and E.*** Larger stromal nodules show greater deposition of matrix and project fully into the lumen of collapsed atrophic acini. ***Panel F***. In certain cases, a collagenous micronodule (asterisk) appears adjacent to regions of transgene expression in the epithelial layer (arrowheads: IHC for HA epitope). ***Panel G.*** Focal immunoreactivity for HA epitope can be observed in epithelium adjacent to regions of periacinar stromal thickening in transgenic mice. ***Panel H.*** A significant elevation in general fibroplasia is noted in transgenic mice over one year of age as compared to age-matched wildtype controls and transgenic mice under one year of age (* *P*<0.05). Images A–F captured at ×200 magnification and image G at ×400.

Fibroplasia was observed in inter-acinar regions in transgenic mice. Similar to other phenotypic observations, the appearance of inter-acinar stromal fibroplasia was evident in transgenic mice over a year of age and consisted of more focal rather than widespread involvement. In regions of focal fibroplasia (Figure 4A) the stroma adjacent to acini exhibited a reactive phenotype associated with elevated deposition of tenascin-C (Figure 4B), a prototypical marker of reactive stroma in the prostate gland as we have reported previously [4], [5]. Some areas of fibroplasia appeared nodular with lamellar deposition of tenascin-C at the base and stalk as well as the periphery of the nodules that extend into the acini lumen (Figures 4D and 4E). The matrix of these stromal nodules was positive in Masson's trichrome (Figure 4F) indicating a deposition of collagen fibrils typically associated with wound repair and fibrosis. The lesions were also similar in histopathology to collagenous micronodules described in human prostate cancer and extended into collapsed glandular acini with attenuated epithelium (Figures 5A–5G). A significant increase in general fibroplasia was noted in mice over a year of age and was significantly higher in transgenic mice as compared to age-matched control mice (Figure 5H). Moreover, transgenic mice exhibited a significant increase in the frequency of the collagenous micronodules as compared to wild type littermates.

### Expression of HA-TGF-β1(a) induces elevated recruitment of monocytes

**Figure 6:**
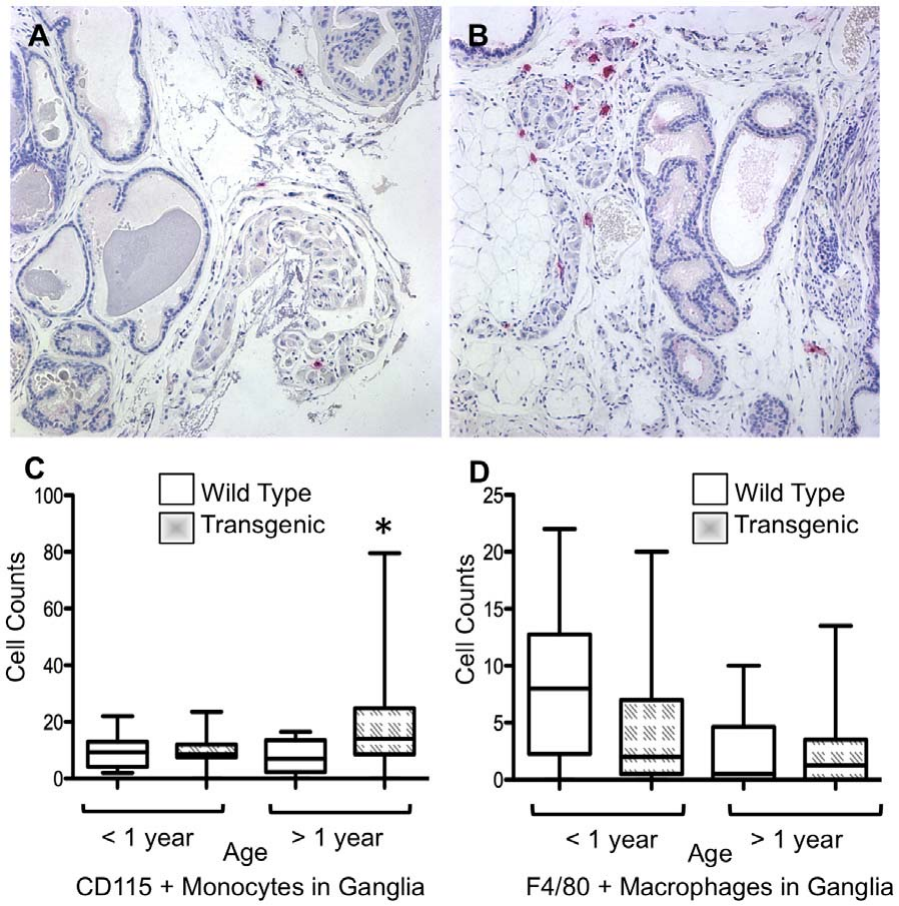
Recruitment of immature myeloid cells to parasympathetic ganglia is increased in TGF-β1 transgenic mice. ***Panels A and B.*** Inflammatory lesions demonstrate significantly greater recruitment of CD115+ myeloid cells to parasympathetic neurovascular bundles in transgenic mice (***Panel B***) compared to wildtype control (***Panel A***). ***Panels C and D***. Quantitation yields significantly greater CD115+ monocytes in transgenic compared to wildtype parasympathetic ganglia in mice over one year of age (* *P*<0.05) (***Panel C***), whereas no significant differences in F4/80 staining is observed (***Panel D***). All images captured at ×200.

Consistent with age-associated inflammatory changes, transgenic mice exhibited differential immunoreactivity of CD115+ myeloid cells in the neurovascular bundles and ganglia compared to wild-type littermates (Figures 6A and 6B). Quantitation revealed a significant increase in the recruitment of CD115+ myeloid cells to nerve ganglia of aged transgenic mice compared to wildtype (Figure 6C). Interestingly, IHC for F4/80 positive macrophages suggested altered immunoreactivity in neurovascular bundles of both wildtype and transgenic mice over one year of age, however quantitation of this trend did not yield statistical significance (Figure 6D). Similarly, there were no significant quantitative changes in either the monocyte or mature macrophage populations within the general stroma of the ventral prostate lobe proper (data not shown).

### Expression of HA-TGF-β1(a) in prostate tissue recombinants produces a fibroplasia phenotype

**Figure 7:**
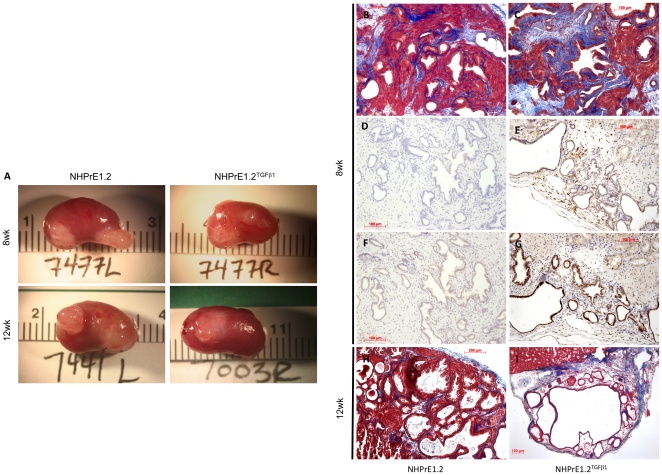
Tissue recombinants demonstrate TGFβ1-induced extracellular matrix deposition and inflammation. ***Panel A.*** Recombination of UGM plus NHPrE1.2 vs. NHPrE1.2-HA-TGF-β1(a) demonstrates a decrease in graft size at both 8 and 12 weeks in recombinants that express HA-TGF-β1(a). ***Panels B and C.*** Trichrome staining demonstrates increased collagen production in HA-TGF-β1(a) expressing human prostate epithelia compared to vector control. ***Panels D and E.*** An increase in F4/80 positive macrophages is apparent in recombinants expressing HA-TGF-β1(a). ***Panels F and G.*** In addition, p65 activation is observed in both the epithelium and stroma in recombinants expressing HA-TGF-β1(a). ***Panels H and I.*** Control recombinants at 12 weeks maintain glandular acini structure and overall morphology, whereas recombinants made with HA-TGF-β1(a) epithelium exhibit atrophic and attenuated acini. Images B-I captured at ×100.

To address HA-TGF-β1(a) in a human epithelium/rodent stroma recombinant model and to further address induced stromal fibroplasia, normal human prostate NHPrE1.2 epithelial cells were engineered to express HA-TGF-β1(a). Control NHPrE1.2 and HA-TGF-β1(a) expressing NHPrE1.2^TGFβ^ cell lines were recombined with rat urogenital mesenchyme (rUGM) and placed under the kidney capsule of SCID mice for 8 or 12 weeks. Tissue recombinants constructed with HA-TGF-β1(a) expressing cells demonstrated a decrease in overall graft size over both time periods (Figure 7A). Both control and experimental recombinants showed that UGM induced NHPrE1.2 cells to differentiate to glandular epithelial acini with associated stroma (Figures 7B and 7C). Compared to controls, however, 8-week tissue recombinants made with HA-TGF-β1(a) expressing NHPrE1.2 cells showed demonstrable increases in collagen deposition, evident by trichrome staining (Figures 7B and 7C) and elevated macrophage infiltration (Figures 7D and 7E). Immunohistochemistry for the p65 subunit of NF-κB corroborated the presence of a macrophage-rich inflammatory infiltrate. Control tissue exhibited essentially no nuclear p65 immunoreactivity (Figure 7F), whereas an increase of p65 activation in both epithelia and a subset of stroma was observed in recombinants made with HA-TGF-β1(a) expressing epithelial cells (Figure 7G). Compared to controls (Figure 7H), 12-week recombinants were either very small or showed severe attenuation of glandular architecture and very limited numbers of attenuated acini (Figure 7I). Accordingly, HA-TGF-β1(a) expression in human prostate progenitor epithelia in tissue recombinations produced a response that was fully consistent with the increases in collagen deposition, inflammatory cell infiltration, and glandular attenuation observed in the HA-TGF-β1(a) transgenic animals.

## Discussion

Data presented here shows that targeted expression of constitutively active TGF-β1 in prostate gland epithelium resulted in focal expression of the transgene that was associated with attenuation and breakdown of the glandular acini wall epithelium, degeneration of secretory acini, inflammation of vessels and nerve ganglia, and an induced stromal fibroplasia. Of interest, these phenotypes evolved as an age-dependent process with significant differences observed in mice over a year of age. The earliest focal phenotype was an attenuation of epithelial cells in acini walls, concomitant with a degeneration of basal lamina integrity in some but not all acini wall regions. Inflammatory and fibroplasia alterations appeared to be secondary to this focal epithelial attenuation. The appearance of pyknotic nuclei in conjunction with focal regions of severe epithelial wall thinning suggested cell damage. Over time, this process of epithelial disruption would be expected to generate a physical breach in the basement membrane thereby providing a gateway of entry for constitutively active TGF-β1 to enter the stromal compartment. Indeed, collagen IV immunohistochemistry showed focal regions of discontinuity in the basal lamina surrounding several ventral prostatic acini in transgenic mice. This is a key alteration in homeostasis since the stroma is normally restricted from access to proteins secreted by epithelium into the lumen within glandular tissues. Such an alteration would also be expected to generate host responses in the stromal compartment. As TGF-β1 is a potent inducer of reactive stroma, it is likely that a repair response was induced by active HA-TGF-β1(a) subsequent to release from damaged acini and loss of the epithelium integrity. This defect is conceivably more pronounced in mice over one year of age owing to both the natural aging processes and TGF-β1-induced accumulated defects in epithelial wall integrity. Our previous reports have shown that reactive stroma initiates adjacent to preneoplastic prostatic intraepithelial neoplasia (PIN) lesions in a heterogeneous pattern where epithelial cells had lost polarity, suggesting defects in acini wall integrity [4]. This study also showed that overexpression of TGF-β1 was first noted in a heterogeneous focal pattern during the evolution of PIN. Data reported here suggests that both the overexpression of TGF-β1 together with the loss in epithelial polarity in PIN results in a focal induction of reactive stroma. These observations are critical to understanding the role of the microenvironment in the clinical progression of prostate cancer. We have shown previously that reactive stroma promotes prostate cancer progression via induction of angiogenesis and the downstream actions of TGF-β1 induced factors in reactive stroma including connective tissue growth factor (CTGF) and fibroblast growth factor 2 (FGF-2) [6], [18], [19]. Moreover, we have reported previously that patients with high grade reactive stroma, exhibit a significantly reduced time to biochemical recurrent disease as evidenced by rise in serum PSA levels [25].

A novel finding in this study was inflammation associated primarily with the neurovascular bundles and local ganglia observed in nearly all transgenic mice over 1 year of age. Both prostate cancer and benign prostatic hyperplasia are associated with elevated inflammation and both exhibit overexpression of TGF-β1 in epithelial cells [3], [4]. Data here is also consistent with the chemokine function of TGF-β1 in recruitment of monocyte/macrophages, however our data shows that recruited cells were primarily monocytes and not mature macrophages. These data would support the concept that TGF-β1 functions primarily to recruit monocyte cell populations but limit their maturation to macrophages. The significance of this tropism for nerve ganglia and how it may affect prostate gland function is not yet understood. Although previous studies using a xenograft tissue recombination model have shown that overexpression of TGF-β1 induced an increased density of phenotypically distinct nerve “ganglion-like cells” [26], the potential role of prostate ganglia inflammation in reactive stroma induction of this transgenic model is not yet understood.

The function of the collagenous micronodules reported here has not been elucidated. One interpretation is that these micronodules evolve as a component of wound repair to induce a collapse of the epithelial acini in damaged, non-functional glands in order to effectively take these acini off-line as a compensatory response. Closure of damaged acini might prevent retrograde introduction of pathogens from the downstream urogenital tract into the prostate gland stroma that houses vessels. Accordingly, in conjunction with the other induced inflammatory responses in vessels and nerves, these nodules may form to ostensibly sequester or limit a potential nidus of infection from spreading in a prostate gland with damaged prostatic acini and epithelial walls with breached integrity. Indeed, these lesions are nearly identical in histopathology to collagenous micronodules associated with human prostate cancer [27], however the function of these nodules has not yet been reported. Furthermore, we have observed similar lesions associated with prostate carcinoma in the Hi-Myc transgenic mouse model (data not shown). Accordingly, we propose here that these nodules form and function as a key component of adaptive homeostasis in glandular tissue to protect from further damage and to promote repair.

The generation of focal fibroplasia, inflammation and collagenous micronodules in the stromal microenvironment of TGF-β1 overexpressing mice is consistent with the role of reactive in wound repair biology [1], [2], [28]. These important adaptive responses whereby fibrosis and inflammation act in coordinate manners may provide additional insight into the tumor promoting nature of the reactive stroma microenvironment observed in most carcinomas [3]. Overexpression of TGF-β1 in other murine epithelial organ systems produces fibrotic responses, albeit somewhat different from what we report here, since inflammation of ganglia and the induction of collagenous micronodules have not been reported previously [13], [14], [16]. Understanding these responses and mechanisms is important for developing novel therapeutic targets for disorders where the stromal microenvironment plays a pivotal role in progression and clinical outcome. Crosses of this HA-TGF-β1(a) mouse into other transgenic backgrounds or use in other experimental conditions may aid in studies of how inflammation and reactive stroma in the microenvironment affects benign, neoplastic, or inflammatory prostate diseases. Further insight into downstream pathways of TGF-β1 and mechanisms in prostate tissue homeostasis is important for understanding the role of this factor in prostate disease progression.
